# HIV-DNA in the Genital Tract of Women on Long-Term Effective Therapy Is Associated to Residual Viremia and Previous AIDS-Defining Illnesses

**DOI:** 10.1371/journal.pone.0069686

**Published:** 2013-08-21

**Authors:** Thierry Prazuck, Antoine Chaillon, Véronique Avettand-Fènoël, Anne-Laure Caplan, Collins Sayang, Aurélie Guigon, Mohamadou Niang, Francis Barin, Christine Rouzioux, Laurent Hocqueloux

**Affiliations:** 1 Department of Infectious Diseases, CHR d'Orléans-La Source, Orléans, France; 2 Laboratory of Virology, CHU Tours, France; 3 Laboratory of Virology, Necker Hospital, EA 3620 Université Paris Descartes, Paris, France; 4 COREVIH, HIV-AIDS Coordinating Units, Centre-Poitou-Charentes, France; 5 Laboratory of Virology, CHR d'Orléans-La Source, Orléans, France; National AIDS Research Institute, India

## Abstract

**Objectives:**

To assess the impact of long-term combined antiretroviral therapy (cART) on HIV-RNA and HIV-DNA levels in cervicovaginal secretions of HIV-1-infected women with sustained undetectable plasma RNA viral load (PVL); to explore factors predictive of residual viral shedding; and to evaluate the risk of heterosexual transmission.

**Methods:**

Women with undetectable PVL (<50 copies/mL) for >6 months were included in this cross-sectional study. HIV-RNA and HIV-DNA were measured in blood and cervicovaginal lavage fluid (CVL). Women were systematically tested for genital infections. The risk of transmission to male partners during unprotected intercourse was estimated.

**Results:**

Eighty-one women composed the study population: all had HIV-RNA <40 copies/mL in CVL. HIV-DNA was detectable in CVL of 29/78 patients (37%). There was a weak positive correlation between HIV-DNA levels in PBMCs and CVL (r = 0.20; p = 0.08). In multivariate analysis, two factors were associated with HIV-DNA detection in CVL: previous AIDS-defining illnesses (OR = 11; 95%CI = 2–61) and current residual viremia (20<PVL<50 cp/mL) (OR = 3.4; 95%CI = 1.1–10.9). Neither the classes of cART regimen nor the presence of genital bacterial or fungal colonization were associated with HIV-DNA detection in CVL. Twenty-eight percent of the women had unprotected intercourse with their regular HIV-seronegative male partner, for between 8 and 158 months. None of their male partners became infected, after a total of 14 000 exposures.

**Conclusion:**

In our experience, HIV-RNA was undetectable in the genital tract of women with sustained control of PVL on cART. HIV-DNA shedding persisted in about one third of cases, with no substantial evidence of residual infectiousness.

## Introduction

It is important to determine the conditions in which unprotected sexual intercourse carries a negligible risk of HIV transmission, both for prevention and for establishing guidelines. Several studies have shown that plasma HIV-RNA suppression by combined antiretroviral therapy (cART) is associated with a huge reduction in the risk of sexual HIV transmission in serodifferent couples [1]–[3]. In January 2008 the Swiss Federal AIDS Commission stated that HIV-infected people on effective cART without other sexually transmitted diseases may be considered “sexually noninfectious” [4]. Subsequent French recommendations considered that unprotected sex was a possible alternative to medically assisted reproduction under the same conditions [5]. Nevertheless, small amounts of HIV-RNA and/or HIV-DNA are frequently present in the genital tract of women on cART, even with recent cART regimens that regularly achieve plasma HIV-RNA viral loads (PVL) below 50 cp/mL [6]–[9]. In cervicovaginal secretions (CVS), cell-free HIV-RNA viral load is the best predictor of the risk of sexual transmission, and cell-associated HIV-DNA is also a marker of potential infectiousness [10], [11]. The presence of HIV-DNA corresponds to the detection of infected cells (i.e. leukocytes) in the genital tract. The level of HIV-DNA may reflect the overall level of HIV infection in the body and/or be a consequence of the persistence of local residual inflammation, which may be maintained or triggered by a bacterial, viral or fungal infection. In addition, the slightest distribution of some antiretroviral drugs in the genital tract, which might contribute to maintain productive infected cells, can lead to a viral compartmentalization. In principle, therefore, the presence of at least one of these markers would imply the need for sexual abstinence and/or systematic condom use, ruling out “normal” sexuality and reproduction. To our knowledge, the residual risk of HIV transmission to male partners of women on long-term effective cART has not been studied in terms of both HIV-RNA and HIV-DNA levels in the genital tract.

The aims of this study were to determine the amounts of HIV-RNA and HIV-DNA in the genital tract of women on long-term effective cART, to identify factors predictive of residual viral shedding in the genital tract, and to assess the risk of sexual transmission to their male partners.

## Patients and Methods

### Population and study design

This was a cross-sectional study. We recruited consecutively non-pregnant HIV-1-infected female outpatients aged 18 years or more who were attending the Department of Infectious Diseases of Orleans Regional Hospital (France) for scheduled routine cervical dysplasia/cancer screening. Women on cART who had had PVL levels below 50 copies/mL for at least 6 months and who had no genital symptoms were invited to join the study. Patients experiencing more than one blip (PVL 50–200 cp/mL framed by PVL <50 cp/mL) per year were excluded. Patients were asked about their adherence to treatment during the past 3 months, and were asked to avoid sexual intercourse, douching and the use of intravaginal procedures or inserts during 48 hours before the study visit.

A “viremic” group of HIV-1-infected women with PVL >100 copies/mL, with or without cART, was also recruited in order to validate the biological methods.

All the participants gave their written informed consent, and the study protocol was approved by our institutional ethics committee (Comité d'éthique Recherche du Centre Hospitalier Régional d'Orléans). Counselling to avoid unprotected sex was systematically provided to study participants.

### Sample collection

Blood and genital samples were collected on the same day, between the 10th and 20th days of the menstrual cycle to avoid contamination by menses. The same practician collected all genital samples throughout the study.

Blood analyses included T cell counts, PVL, HIV-DNA quantification in peripheral blood mononuclear cells (PBMCs), and syphilis serology. Urine was systematically tested for *Chlamydia trachomatis* by PCR (Real Time CT, Abbott).

Genital specimens were collected after careful visual examination, using a speculum. First, vaginal swabbing was performed to screen for fungal and bacterial infections, by wet mount, Gram staining, white and red blood cell counts, and culture in appropriate media (including medium specific for *Neisseria spp*). Yeast cells were detected by light microscopy on a saline wet preparation and were identified by direct examination. Bacterial vaginosis was diagnosed with the Nugent score [12]. CVS were then collected for viral assays. Specimens were obtained by vaginal lavage (douching) with 6 mL of phosphate-buffered saline inserted into the vagina, left to pool for 1 min, then reaspirated and re-inserted 3 to 5 times, as described by Belec *et al* [13]. Cervicovaginal lavage fluid (CVL) was immediately stored at −80°C for pooled analysis. An additional swab was collected for Y chromosome detection in CVL of women with detectable vaginal VL, in order to rule out contamination by an HIV-positive male partner. Finally, cervical cytology was performed.

### Virological methods

#### Quantification of HIV-1 RNA in blood and CVS

Plasma and vaginal HIV-RNA viral loads were determined by RT-PCR, using the Abbott RealTime HIV-1 assay, as recommended by the manufacturer (Abbott Molecular Inc., Des Plaines, IL, USA, 2007), in 1 ml of each sample. The positivity cut off was 40 copies/mL for both blood and genital samples. The study group was subdivided around the sensitivity cut off of the Abbott RealTime assay, into a group with undetectable PVL (<20 cp/mL) and a group with residual viremia (RV) (20<PVL<40 cp/mL). Because most current guidelines and recent reports consider <50 cp/mL as the standard threshold for “undetectability”, we integrated PVL values between 40 and <50 cp/mL in the RV group.

#### Quantification of cell-associated HIV-DNA in blood and CVS

HIV-DNA was extracted from 200 µL of blood and 1 mL of CVL, using the Nucleospin blood kit (Macherey-Nagel). Total cell-associated HIV-DNA was quantified in these extracts by using the ANRS ultrasensitive real-time PCR method (Biocentric, Bandol, France), as previously described (amplification of the LTR region), in quadruplicate [14]. Results were expressed as log_10_ copies per million PBMCs and as log_10_ copies per million vaginal cells.

#### Detection of herpes simplex virus type 2 (HSV-2) DNA in CVS

HSV-2 DNA was detected using a qualitative, real-time PCR assay (HSV2 R-geneTM, Argene Inc., NY, USA) (detection threshold: 50 copies/mL).

### Estimation of sexual exposure among male partners

Women in the study group were interviewed using a structured questionnaire that included condom use with stable male partners. When a woman said she had unprotected intercourse, the latest HIV serologic status of her partner(s) was collected. The partners were re-tested if their last test was done more than 3 months before the study day. The stated monthly frequency of sexual intercourse and the total duration of sexual exposure of unprotected male partners were used to estimate the total number of unprotected sexual acts during the years preceeding the study, by multiplying the monthly frequency of intercourse by the time until the last negative HIV test. Only the period during which the women were on cART was considered in these analyses (i.e.: we excluded from analysis all cases where the male partner was known to be seropositive for HIV before cART was started in his partner).

### Statistical analyses

Categorical data were compared with Fisher's exact test or the χ^2^ test, and continuous variables with the Kruskall-Wallis or Mann-Whitney U test. The association between outcomes of interest and various factors, including all demographic and immuno-virological data at baseline, were tested in a multivariate logistic regression model. Linear correlations were analyzed with Pearson's test. MedCalc statistical software (MedCalc Software, Mariakerke, Belgium) was used for all analyses.

## Results

### Population characteristics

Ninety-seven women were enrolled, 81 in the study group (PVL <50 cp/mL) and 16 in the viremic group (PVL >100 cp/mL). Overall, two patients only declined to join the study. In the study group, the median duration of PVL <50 cp/mL was 44 months [IQR: 21–68]: among them 58 patients (72%) had PVL <20 cp/mL and 23 (28%) had residual viremia (20<PVL<50 cp/mL) at the time of inclusion. The viremic group comprised 9 cART-naïve patients and 7 patients with detectable PVL on cART. The clinical and virological characteristics of the patients are summarized in Table 1.

**Table 1:** Baseline characteristics of women included in the study group (n = 81) and viremic group (n = 16).

		Study group	Viremic group
		(n = 81)	(n = 16)
Age, years		40	35,5
		[35]-[50]	[30]-[50]
Ethnicity, n (%)			
	Sub-Saharan African	58 (72)	12 (75)
	European	19	4
	Other	4	0
Heterosexual HIV transmission, n (%)		74 (91)	16 (100)
CDC staging, n			
	A	49	12
	B	16	2
	C	16	2
Co-infection, n			
	none	68	14
	HBV	6	2
	HCV	7	0
Lowest CD4+ T cell count, /µL		230	303
		[147-305]	[116-423]
Highest plasma HIV-RNA, log/mL		5.1	4.8
		[4.6-5.6]	[3.9-5.7]
Plasma HIV-RNA, log/mL		<1.7	3.7
			[3.3-4.8]
Vaginal HIV-RNA, log/mL		<1.6	2.2
			[<1.6-2.4]
HIV-DNA in blood, log/10^6^ PBMCs		2.7	2.8
		[2.4-3.1]	[2.4-3.1]
HIV-DNA in vagina, cp/10^6^ cells		0	0
		[0-6]	[0-11]
CD4+ T cell count, /µL		663	499
		[467-889]	[316-627]
% CD4+ T cells		33	24
		[27]-[40]	[18]-[36]
CD4+/CD8+ ratio		0.9	0.5
		[0.6-1.3]	[0.3-0.9]
Vaginal colonization, n (%)		24 (30)	11 (69)
median [IQR]			

### Blood and genital HIV-RNA levels

**Figure 1:**
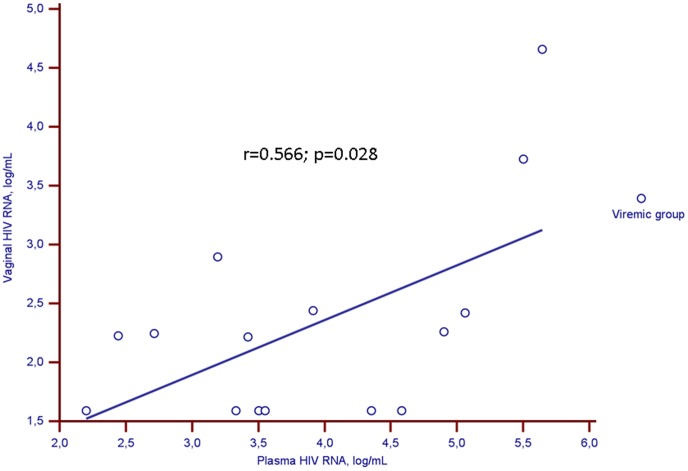
Correlation between HIV-RNA viral loads in paired plasma and cervicovaginal samples in the viremic group. Circles represent women with plasma HIV-RNA viral load >100 cp/mL (viremic group). The solid line is the regression line for this population.

In the study group, all 81 women with sustained PVL <50 cp/mL had CVL HIV RNA levels below 40 copies/mL. In the viremic group, the median VL was 3.7 log_10_ cp/mL [3.3–4.8] in plasma and 2.2 log_10_ cp/10^6^ cells [<1.6–2.4] in CVL. We found a strong positive correlation between HIV-RNA levels in plasma and CVL (r = 0.566; p = 0.028) (Fig. 1). The median HIV-RNA level was at least 10 times higher in plasma than in CVL.

### Blood and genital HIV-DNA levels

**Figure 2:**
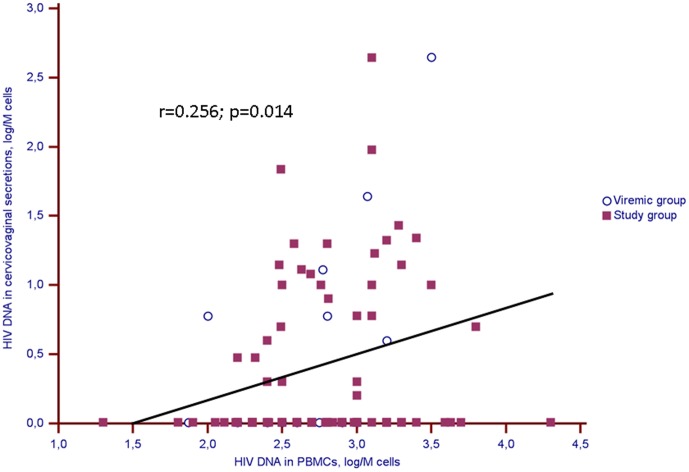
Correlation between HIV-DNA levels in paired blood and genital samples in the whole population (study and viremic groups). Solid squares represent women with plasma HIV-RNA viral load <50 cp/mL (study group), and circles women with plasma HIV-RNA viral load >100 cp/mL (viremic group). The solid line is the regression line for the whole population.

HIV-DNA levels in PBMCs and vaginal cells were similar in the study and viremic groups (Table 1). There was a weak positive correlation between PBMCs and vaginal cells-associated HIV-DNA levels in the overall population (r = 0.26; p = 0.01) (Fig. 2), and also in the study group (r = 0.20; p = 0.08) and the viremic group (r = 0.57; p = 0.03).

HIV-DNA was detected in vaginal cells of 29/78 patients (37%) in the study group and 7/15 patients (47%) in the viremic group (p = 0.6). In the study group, HIV-DNA was detected in vaginal cells of 12/33 patients with RV and 17/55 of those with PVL<20 cp/mL (p = 0.12). In patients with RV the median HIV-DNA level in vaginal cells was 2 cp/10^6^ cells [IQR = 0–10] versus 0 cp/10^6^ cells [IQR = 0–3] in those with PVL<20 cp/mL (p = 0.097).

### Microbiology findings in the genital tract

In the study group, although the patients all denied genital symptoms, 29/81 (36%) had at least one laboratory-diagnosed vaginal colonization: 17 (21%) had bacterial vaginosis and 8 (9%) had vaginal candidiasis (1 had dual colonization) (Table 1 and 2). No cases of *Trichomonas*, *N. gonorrhoeae* or *C. trachomatis* vaginal infection were found. None of the women self-declared genital ulcers and none had evidence of ulceration on visual inspection. Only one patient had asymptomatic HSV-2 shedding.

**Table 2:** Analysis of characteristics associated with HIV-DNA detection in the genital tract of women included in the study group.

		Undetectable	Detectable		
		HIV-DNA in vagina	HIV-DNA in vagina	*P* value	*P* value
		(n = 49)	(n = 29)	(univ.)	(multiv.)
Age, years		40	38,0	0.18	
		[35]-[52]	[34]-[44]		
Ethnicity, n				0.08	
	Sub-Saharan African	34	21		
	European	14	5		
	Other	1	3		
Heterosexual HIV transmission, n (%)		45 (92)	26 (90)	0.6	
CDC staging, n				0.009	
	A	37	12		
	B	7	8		
	C	5	9		0.006
Co-infection, n				0.3	
	none	42	23		
	HBV	2	4		
	HCV	5	2		
Lowest CD4+ T cells, /µL		18	12	0.005	
		[12]-[26]	[8]-[16]		
Highest plasma HIV-RNA, log/mL		4.9	5.4	0.06	
		[4.6-5.4]	[4.8-5.7]		
Time from HIV diagnosis to treatment, y		0.4	0.5	0.9	
		[0.1-3]	[0.1-3.1]		
CD4+ T cell count, /µL		709	613	0.15	
		[496-894]	[408-800]		
% CD4+ T cells		36	30	0.009	
		[29]-[41]	[25]-[37]		
CD4+/CD8+ ratio		1	0.7	0.02	
		[0.7-1.4]	[0.6-1.1]		
Residual viremia, n (%)		11 (22)	12 (41)	0.13	0.04
HIV-DNA in blood, log/10^6^ PBMCs		2.7	2.8	0.13	
		[2.3-3]	[2.5-3.1]		
Time with plasma HIV-RNA <50 cp/mL, mo		48	36	0.17	
		[23-72]	[16-65]		
cART regimens, n				0.5	
	3 NRTIs	3	1		
	2 NRTIs + 1 NNRTI	20	11		
	2 NRTIs + 1 PI	24	14		
	2 NRTIs + 1 II	1	3		
Vaginal colonization, n (%)		12 (24)	10 (34)	0.5	
White blood cells/field, n		40	10	0.08	
		[0-400]	[0-50]		
Vaginal cells/field, n		100	70	0.3	
		[43-188]	[43-100]		
median [IQR]					

Abbreviations : **HBV: hepatitis B virus; HCV: hepatitis C virus; II: integrase inhibitor; NRTI: nucleoside/nucleotide reverse transcriptase inhibitor; NNRTI: non nucleoside reverse transcriptase inhibitor; PI: protease inhibitor.**

### Factors associated with the presence of HIV-DNA in vaginal cells of patients in the study group

In univariate analysis, the presence of HIV-DNA in CVL was associated with the CDC stage, the CD4+ nadir, the current %CD4+, and the current CD4+/CD8+ ratio (Table 2). There was no correlation between the CVL HIV-DNA level and the duration of undetectable PVL, vaginal colonization, or the class of cART regimen.

In multivariate analysis, only a history of AIDS-defining illness (OR = 11; 95%CI = 2–61) and current residual viremia (OR = 3.4; 95%CI = 1.1–10.9) were associated with an increased risk of HIV-DNA detection in vaginal cells.

### Estimated risk of HIV transmission to male partners

All 81 women in the study group said they had only one, stable partner, and 23 of them (28.4%) said they routinely had unprotected intercourse. These women said they had sex 13 times a month on average, over a mean study period of 46 months [range: 8–132 months], yielding an estimated 14 000 exposures among their partners. Genital HIV-DNA was detected in 8 (34.8%) of the women who had unprotected sexual intercourse (median: 10 cp/10^6^ cells [range: 6–442]). All of these women had CD4+ cell counts above 500/mm^3^ and half of them had residual plasma viral load. Overall, none of the male partners was found to be HIV positive at the last test they underwent.

## Discussion

This is one of the largest investigation of genital HIV-1 shedding (including both HIV-RNA and HIV-DNA) among women on long-term effective cART in an industrialized country. Another strength of this study is that the male partners' HIV serostatus was also determined. We found that HIV-RNA was undetectable in the genital tract of women who had sustained plasma viral load below 50 copies/mL on cART, regardless of the drugs used, and that none of their male partners became infected during the study period. However, a low level of HIV-DNA was frequently detected in these women's cervicovaginal secretions, raising the possibility of residual infectiousness. Noteworthy, we found that the detection of HIV-DNA in the genital tract was statistically linked to the presence of a residual viremia.

We found that 39.5% of women with undetectable PVL had detectable HIV-DNA in the genital tract, a rate very similar to those previously reported (median 34%, IQR = 30–40) [8]–[10], [15]–[17]. Overall, there was a positive correlation between HIV-DNA shedding in the genital tract and the level of HIV-DNA in PBMCs, although this correlation was less pronounced than the one we previously found between gut-associated lymphoïd tissue and blood [18]. Noteworthy, one of the two factors independently associated with HIV-DNA shedding in the genital tract was a residual viremia. A low level of plasma HIV-RNA could be a marker of ongoing replication in HIV sanctuaries, particularly the genital tract where some antiretroviral drugs diffuse poorly [19], [20]. The infectiousness of HIV-DNA-containing vaginal cells is unclear. Baeten *et al* reported cases of HIV transmission among male partners of untreated women with undetectable HIV-RNA in CVS [11]. HIV-DNA in CVS could also be a surrogate marker for residual genital HIV-RNA transcription below the detection limit of current methods.

Although there is a good correlation between HIV-RNA viral load in paired blood and genital samples, it was recently reported that genital HIV-RNA shedding is predictive of heterosexual HIV transmission, independently of the plasma HIV-RNA concentration [11]. Prospective studies have shown that effective cART rapidly reduces both HIV-RNA and HIV-DNA shedding in CVS, but whether or not this persists over several years has rarely been investigated [8]–[10], [16], [17]. All the patients studied here were taking one of the optimized cART regimens currently used in Europe, based on systematic genotyping; in addition, self-reported adherence was excellent (data not shown) and follow-up took place in optimal conditions, contrary to previous studies in resource-poor settings [21]. Likewise, few protease inhibitor (PI)-based regimens are used in most sub-Saharan countries, while PI appear to be more effective than NNRTI in suppressing genital HIV-RNA shedding [6], [17], [22]–[24]. Finally, pretreatment HIV genotyping is not routinely used in poor countries. This could lead to lower efficacy in case of primary resistance, particularly in countries where nevirapine monotherapy is generally used to prevent mother-to-child transmission [25]. Of note, we found no association between the class of cART (in particular PI vs. NNRTI) and genital viral shedding.

One important limitation of this study is its cross-sectional nature. The results obtained here do not rule out the possibility that HIV-RNA is occasionally shed in the genital tract of HIV-infected women with undetectable PVL, as reported elsewhere [7], [9]. It is noteworthy that we found lower rates of viral shedding in CVS than previously reported [6]–[10], [15]–[17], [23], [26]. However, most previous studies included patients who had intermittent viremia and/or a shorter duration of undetectable PVL. One another limitation is the sampling method we used. Indeed, we performed cervicovaginal lavage prior to any potentially traumatic procedure, as the presence of blood could interfere with shedding measurements. CVL also increases the sampling area and collects a large volume of fluid that can be fractionated for analysis. However, this method leads to dilution of viral particles. In a previous study including untreated viremic women, HIV-1 RNA was detected in respectively 57%, 61% and 79% of vaginal tampons, CVL, and cervicovaginal lavage fluid samples enriched with a cervical swab (eCVL) [27]. In an adjusted analysis excluding samples containing microscopic traces of blood, CVL was still 1.4 times (95% CI 1.05 to 1.88; P = 0.022) less sensitive than eCVL [27]. Although less sensitive than enriched lavage, the CVL method more closely mimics natural conditions of HIV-1 transmission and avoids blood contamination [27]. The long period of PVL suppression and the potent cART regimens used here could also have contributed to the low frequency of HIV-RNA detection. Interestingly, the frequency of HIV-RNA detection in the genital tract of women on cART has tended to fall during the last decade: in 6 studies conducted up to 2007, a median 27% (IQR = 25–30) of women had positive results, while this rate was only 14% (IQR = 11–15) in 4 studies conducted between 2010 and 2011 [6]–[10], [15]–[17], [23], [26].

Additional factors such as incomplete adherence to therapy and active sexually transmitted infections may increase the risk of sexual transmission [23], [28]–[30]. Some intravaginal practices among African women disrupt the vaginal flora, increase the risk of vaginosis, and are associated with intermittent HIV-RNA shedding in the genital tract [31]. Thus, local inflammation due to intravaginal use of aggressive products, rather than the subsequent vaginosis, could trigger intermittent local HIV-RNA transcription by activated HIV-infected lymphocytes. However, we did not find that genital colonization (bacterial vaginosis or candidiasis) or higher vaginal cell counts were associated with HIV-DNA shedding in the genital tract.

Heterosexual HIV transmission involves interactions between biological and behavioural factors. Thus, it is important to study sexual practices in serodifferent couples. We were surprised that a substantial proportion of the HIV-infected women studied here regularly had unprotected sexual intercourse with their partners, with a cumulative total of 14 000 unprotected exposures. None of the partners was contaminated, in keeping with previous studies in which no HIV infection was noted among serodifferent couples when the index case was on effective cART [1], [2]. Given the limited number of couples, and the observational nature of our study, these results should be considered cautiously. Effective cART has been shown by others to have a strong protective effect on HIV transmission within serodifferent couples [3], but larger studies are still needed to confirm this low risk.

In conclusion, we found that women with long-term suppression of plasma viral load on cART had undetectable HIV-RNA in their cervicovaginal secretions, and that none of their male partners became infected, despite frequently unprotected intercourse. Nevertheless, small amounts of HIV-DNA were detected in half the women, suggesting there may be a low residual risk of sexual transmission. The infectiousness of cell-associated HIV-DNA has to be investigated further.

## References

[1] Attia S, Egger M, Müller M, Zwahlen M, Low N (2009) Sexual transmission of HIV according to viral load and antiretroviral therapy: systematic review and meta-analysis. AIDS 23: 1397–1404 doi:10.1097/QAD.0b013e32832b7dca 19381076 10.1097/QAD.0b013e32832b7dca

[2] Del Romero J, Castilla J, Hernando V, Rodríguez C, García S (2010) Combined antiretroviral treatment and heterosexual transmission of HIV-1: cross sectional and prospective cohort study. BMJ 340: c2205. 20472675 10.1136/bmj.c2205 PMC2871073

[3] Cohen MS, Chen YQ, McCauley M, Gamble T, Hosseinipour MC, et al (2011) Prevention of HIV-1 infection with early antiretroviral therapy. N Engl J Med 365: 493–505 doi:10.1056/NEJMoa1105243 21767103 10.1056/NEJMoa1105243 PMC3200068

[4] Vernazza P, Hirschel B, Bernasconi E, Flepp M (2008) Les personnes séropositives ne souffrant d'aucune autre MST et suivant un traitement antirétroviral efficace ne transmettent pas le VIH par voie sexuelle. Schweiz Arzteztg 89: 165–169.

[5] Yeni P (2010) Prise en charge médicale des personnes infectées par le VIH. Paris: La documentation française.

[6] Neely MN, Benning L, Xu J, Strickler HD, Greenblatt RM, et al (2007) Cervical shedding of HIV-1 RNA among women with low levels of viremia while receiving highly active antiretroviral therapy. J Acquir Immune Defic Syndr 44: 38–42 doi:10.1097/01.qai.0000248352.18007.1f 17106279 10.1097/01.qai.0000248352.18007.1f PMC3126662

[7] Cu-Uvin S, DeLong AK, Venkatesh KK, Hogan JW, Ingersoll J, et al (2010) Genital tract HIV-1 RNA shedding among women with below detectable plasma viral load. AIDS 24: 2489–2497 doi:10.1097/QAD.0b013e32833e5043 20736815 10.1097/QAD.0b013e32833e5043

[8] Henning TR, Kissinger P, Lacour N, Meyaski-Schluter M, Clark R, et al (2010) Elevated cervical white blood cell infiltrate is associated with genital HIV detection in a longitudinal cohort of antiretroviral therapy-adherent women. J Infect Dis 202: 1543–1552 doi:10.1086/656720 20925530 10.1086/656720

[9] Launay O, Tod M, Tschöpe I, Si-Mohamed A, Bélarbi L, et al (2011) Residual HIV-1 RNA and HIV-1 DNA production in the genital tract reservoir of women treated with HAART: the prospective ANRS EP24 GYNODYN study. Antivir Ther (Lond) 16: 843–852 doi:10.3851/IMP1856 21900716 10.3851/IMP1856

[10] Spinillo A, Debiaggi M, Zara F, Maserati R, Polatti F, et al (2001) Factors associated with nucleic acids related to human immunodeficiency virus type 1 in cervico-vaginal secretions. BJOG 108: 634–641. 11426900 10.1111/j.1471-0528.2001.00141.x

[11] Baeten JM, Kahle E, Lingappa JR, Coombs RW, Delany-Moretlwe S, et al (2011) Genital HIV-1 RNA predicts risk of heterosexual HIV-1 transmission. Sci Transl Med 3: 77ra29 doi:10.1126/scitranslmed.3001888 10.1126/scitranslmed.3001888 PMC3087186 21471433

[12] Nugent RP, Krohn MA, Hillier SL (1991) Reliability of diagnosing bacterial vaginosis is improved by a standardized method of gram stain interpretation. J Clin Microbiol 29: 297–301. 1706728 10.1128/jcm.29.2.297-301.1991 PMC269757

[13] Bélec L, Meillet D, Lévy M, Georges A, Tévi-Bénissan C, et al (1995) Dilution assessment of cervicovaginal secretions obtained by vaginal washing for immunological assays. Clin Diagn Lab Immunol 2: 57–61. 7719914 10.1128/cdli.2.1.57-61.1995 PMC170101

[14] Avettand-Fènoël V, Chaix M-L, Blanche S, Burgard M, Floch C, et al (2009) LTR real-time PCR for HIV-1 DNA quantitation in blood cells for early diagnosis in infants born to seropositive mothers treated in HAART area (ANRS CO 01). J Med Virol 81: 217–223 doi:10.1002/jmv.21390 19107966 10.1002/jmv.21390

[15] Shaheen F, Sison AV, McIntosh L, Mukhtar M, Pomerantz RJ (1999) Analysis of HIV-1 in the cervicovaginal secretions and blood of pregnant and nonpregnant women. J Hum Virol 2: 154–166. 10413367

[16] Debiaggi M, Zara F, Spinillo A, De Santolo A, Maserati R, et al (2001) Viral excretion in cervicovaginal secretions of HIV-1-infected women receiving antiretroviral therapy. Eur J Clin Microbiol Infect Dis 20: 91–96. 11305478 10.1007/s100960000442

[17] Graham SM, Holte SE, Peshu NM, Richardson BA, Panteleeff DD, et al (2007) Initiation of antiretroviral therapy leads to a rapid decline in cervical and vaginal HIV-1 shedding. AIDS 21: 501–507 doi:10.1097/QAD.0b013e32801424bd 17301569 10.1097/QAD.0b013e32801424bd

[18] Avettand-Fenoel V, Prazuck T, Hocqueloux L, Melard A, Michau C, et al (2008) HIV-DNA in rectal cells is well correlated with HIV-DNA in blood in different groups of patients, including long-term non-progressors. AIDS 22: 1880–1882 doi:10.1097/QAD.0b013e32830fbdbc 18753926 10.1097/QAD.0b013e32830fbdbc

[19] Kwara A, Delong A, Rezk N, Hogan J, Burtwell H, et al (2008) Antiretroviral drug concentrations and HIV RNA in the genital tract of HIV-infected women receiving long-term highly active antiretroviral therapy. Clin Infect Dis 46: 719–725 doi:10.1086/527387 18220480 10.1086/527387

[20] Taylor S, Davies S (2010) Antiretroviral drug concentrations in the male and female genital tract: implications for the sexual transmission of HIV. Curr Opin HIV AIDS 5: 335–343 doi:10.1097/COH.0b013e32833a0b69 20543610 10.1097/COH.0b013e32833a0b69

[21] Ivers LC, Kendrick D, Doucette K (2005) Efficacy of antiretroviral therapy programs in resource-poor settings: a meta-analysis of the published literature. Clin Infect Dis 41: 217–224 doi:10.1086/431199 15983918 10.1086/431199

[22] Quinn TC, Wawer MJ, Sewankambo N, Serwadda D, Li C, et al (2000) Viral load and heterosexual transmission of human immunodeficiency virus type 1. Rakai Project Study Group. N Engl J Med 342: 921–929 doi:10.1056/NEJM200003303421303 10738050 10.1056/NEJM200003303421303

[23] Graham SM, Masese L, Gitau R, Jalalian-Lechak Z, Richardson BA, et al (2010) Antiretroviral adherence and development of drug resistance are the strongest predictors of genital HIV-1 shedding among women initiating treatment. J Infect Dis 202: 1538–1542 doi:10.1086/656790 20923373 10.1086/656790 PMC2957525

[24] Nagot N, Foulongne V, Becquart P, Mayaud P, Konate I, et al (2005) Longitudinal assessment of HIV-1 and HSV-2 shedding in the genital tract of West African women. J Acquir Immune Defic Syndr 39: 632–634. 16044019

[25] Shekelle P, Maglione M, Geotz MB, Wagner G, Wang Z, et al (2007) Antiretroviral (ARV) drug resistance in the developing world. Evid Rep Technol Assess (Full Rep) 1–74. PMC4781335 18088163

[26] Fiore JR, Suligoi B, Saracino A, Di Stefano M, Bugarini R, et al (2003) Correlates of HIV-1 shedding in cervicovaginal secretions and effects of antiretroviral therapies. AIDS 17: 2169–2176 doi:10.1097/01.aids.0000088178.01779.b4 14523273 10.1097/00002030-200310170-00004

[27] Delany S, Rosas R, Mlaba N, Clayton T, Akpomiemie G, et al (2008) Comparison of cervicovaginal lavage, cervicovaginal lavage enriched with cervical swab, and vaginal tampon for the detection of HIV-1 RNA and HSV-2 DNA in genital secretions. J Acquir Immune Defic Syndr 49: 406–409. 19186353 10.1097/qai.0b013e31818c7f75

[28] Gray RH, Wawer MJ, Brookmeyer R, Sewankambo NK, Serwadda D, et al (2001) Probability of HIV-1 transmission per coital act in monogamous, heterosexual, HIV-1-discordant couples in Rakai, Uganda. Lancet 357: 1149–1153 doi:10.1016/S0140-6736(00)04331-2 11323041 10.1016/S0140-6736(00)04331-2

[29] Wawer MJ, Gray RH, Sewankambo NK, Serwadda D, Li X, et al (2005) Rates of HIV-1 transmission per coital act, by stage of HIV-1 infection, in Rakai, Uganda. J Infect Dis 191: 1403–1409 doi:10.1086/429411 15809897 10.1086/429411

[30] Freeman EE, Weiss HA, Glynn JR, Cross PL, Whitworth JA, et al (2006) Herpes simplex virus 2 infection increases HIV acquisition in men and women: systematic review and meta-analysis of longitudinal studies. AIDS 20: 73–83. 16327322 10.1097/01.aids.0000198081.09337.a7

[31] Low N, Chersich MF, Schmidlin K, Egger M, Francis SC, et al (2011) Intravaginal practices, bacterial vaginosis, and HIV infection in women: individual participant data meta-analysis. PLoS Med 8: e1000416 doi:10.1371/journal.pmed.1000416 21358808 10.1371/journal.pmed.1000416 PMC3039685

